# Pressure Alopecia in a Pediatric Patient: A Case Report

**DOI:** 10.7759/cureus.110607

**Published:** 2026-06-10

**Authors:** Karen Sanchez-Tamayo, Roxana Castañeda-Yépiz, Mariana Pérez-Chavez, Luis Guillermo Patiño-Gutiérrez, Esperanza Sañudo-Vallejo

**Affiliations:** 1 Dermatology, Instituto Dermatológico de Jalisco "Dr. José Barba Rubio", Zapopan, MEX; 2 Dermatology, Del Prado Medical Tower, Tijuana, MEX; 3 Pediatric Dermatology, Instituto Dermatológico de Jalisco "Dr. José Barba Rubio", Zapopan, MEX; 4 Dermatology, Centro Dermatológico de Sinaloa, Culiacán, MEX

**Keywords:** hair diseases, immobilization, pediatric, pressure alopecia, trichoscopy

## Abstract

Pressure alopecia (PA) is a type of secondary alopecia that predominantly affects adults, with only a few pediatric cases reported. We present the case of a four-year-old pediatric patient who developed an alopecic patch after a two-week stay in the intensive care unit and ultimately showed signs of scarring alopecia despite early treatment. Trichoscopy revealed comedone-like dots, which supported this diagnosis. Two months after treatment with steroids and zinc pyrithione shampoo, the patient showed signs of cicatricial alopecia.

Due to the scarcity of reported cases, diagnosis can be challenging and requires a high index of suspicion, particularly in patients with a history of prolonged immobilization associated with hospitalization or surgery. Trichoscopy can assist in distinguishing pressure alopecia from more common differential diagnoses in this age group. Although spontaneous recovery is often observed, early detection is essential for preventing permanent alopecia.

## Introduction

Pressure alopecia (PA) is a rare but significant clinical entity characterized by localized hair loss following prolonged tissue immobilization. While well-documented in adults, its reported incidence remains low, with approximately 100 cases currently recognized [1,2]. The underlying pathophysiology is primarily ischemic; when external pressure on the scalp exceeds capillary perfusion pressure, typically around 32 mmHg, it triggers localized hypoxemia [3]. This metabolic stress can lead to transient follicular shutdown or full-thickness tissue necrosis. Key risk factors associated with this condition include prolonged immobilization, extended operative positioning during lengthy surgical procedures, heavy sedation, and the use of restrictive medical devices [1,2].

Although most reported data on PA derive from adult cohorts, the condition also manifests in the pediatric population, where its clinical profile presents unique diagnostic challenges. Outside of the neonatal period, PA remains an exceptionally rare phenomenon outside of the neonatal period. In fact, most documented cases are confined to the neonatal period, in infants with prolonged stays in the intensive care unit (ICU) [1,4]. Early clinical recognition is essential to the patient's long-term prognosis. This is critical because persistent or unrecognized ischemia can rapidly transition into permanent cicatricial scarring alopecia [4]. We report an unusual case of PA in a four-year-old patient, an age group less commonly affected, who developed the condition three weeks post-discharge following an ICU stay for peripheral neuropathy. This case serves to underscore the necessity of proactive scalp surveillance in all immobilized pediatric patients, regardless of age, to prevent irreversible follicular destruction.

## Case presentation

**Figure 1:**
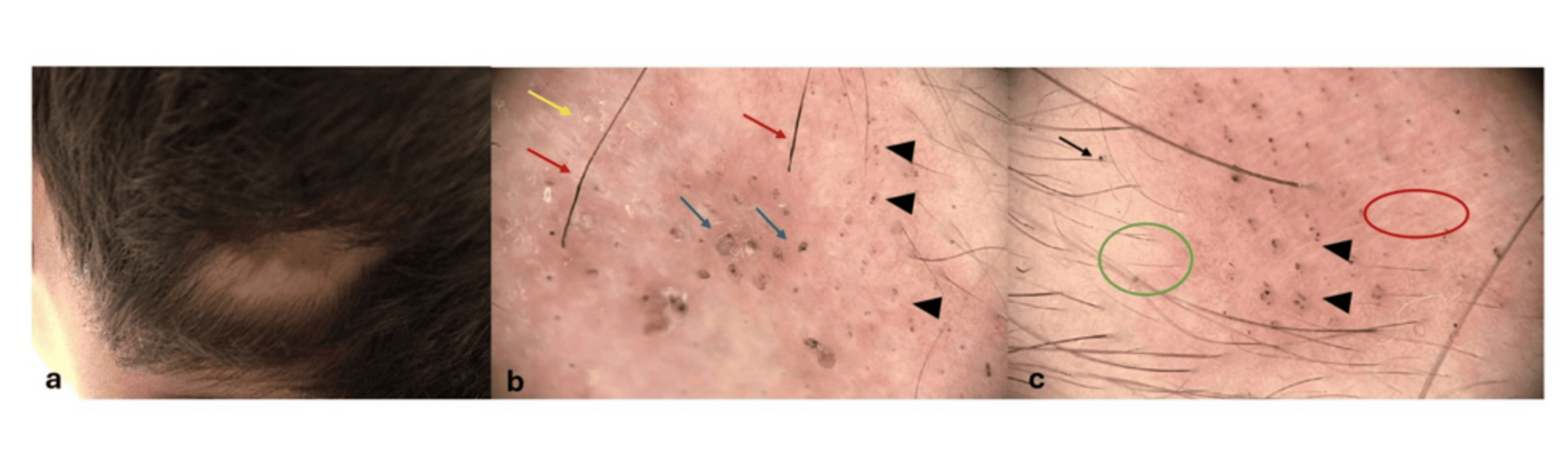
Initial clinical and trichoscopic presentation of occipital pressure alopecia An oval alopecic patch measuring 7x6 cm on the occipital scalp, presenting with a central area of erythematous-violaceous crusts 5 weeks after the initial inciting immobilization event. Initial trichoscopic features demonstrating comedone-like black dots (arrowheads) and follicular plugs (blue arrows), pili torti (red arrows), and whitish interfollicular scales (yellow arrow). High-magnification trichoscopy highlighting vellus hairs (green circle), absent follicular openings (red circle), black dots (black arrow), and comedone-like black dots (arrow heads).

A four-year-old male with a history of a two-week ICU hospitalization for Guillain-Barré syndrome presented three weeks post-discharge with an occipital scalp dermatosis. During his 14-day ICU stay, the patient required continuous immobilization and was maintained in a supine position for most of the time, with positional changes performed every 8 hours. No other systemic or localized risk factors were identified. Five weeks after the onset of immobilization (three weeks following hospital discharge), the patient developed an occipital scalp lesion. Physical examination demonstrated a 7x6 cm oval alopecic patch with well-defined borders and a central area of erythematous-violaceous crusts that was tender to palpation (Figure 1a). Trichoscopy revealed comedo-like black dots (Figure 1b), vellus hairs, absent follicular openings, pili torti, and interfollicular whitish scales (Figure 1c).

Differential diagnosis included alopecia areata (AA) and tinea capitis. Alopecia areata was excluded due to the presence of central crusts, localized tenderness, and the absence of exclamation mark hairs or widespread yellow dots on trichoscopy. Tinea capitis was ruled out based on the direct chronological link to prolonged pressure and the absence of characteristic trichoscopic features, such as corkscrew hairs, Morse code-like hairs, or comma hairs. Consequently, a diagnosis of type 1 PA was established.

**Figure 2:**
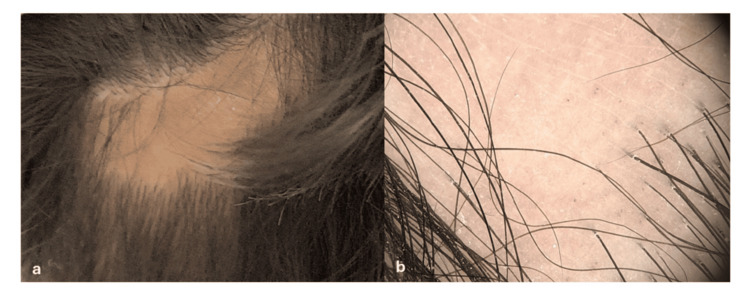
Clinical (a) and trichoscopic (b) followup at one month post-treatment Follow-up demonstrating hair regrowth restricted to the periphery with persistent scarring in the center of the patch.

Initial treatment included zinc pyrithione shampoo to manage the interfolicular scaling and clobetasol propionate 0.05% cream twice a week, prescribed as a potent topical anti-inflammatory to mitigate tissue stress and localized inflammation secondary to ischemia. After one month, an improvement in scaling was observed. The topical steroid dose was tapered to once a week to prevent skin atrophy, and topical minoxidil 5 % was initiated to promote the anagen phase and accelerate hair regrowth in the viable follicles. Follow-up trichoscopy demonstrated hair regrowth only at the periphery of the lesion (Figures 2a-2b).

## Discussion

PA is a secondary alopecia resulting from ischemic events on the scalp, usually caused by sustained external pressure during surgery or ICU stays. In some cases, this can also result from internal pressure from cosmetic injectables [4].

Although the pathogenesis is complex, the core mechanism involves external pressure exceeding capillary hydrostatic pressure, leading to localized tissue hypoxia and cellular distress [5,6]. Abel et al. [7] and Lawson et al. [3] were among the first to report postoperative alopecia, and Lawson proposed a correlation between the duration of the procedure and risk of scarring alopecia. Two types have been proposed: type 1, which is due to external pressure-induced ischemia, and type 2, which is secondary to internal pressure after cosmetic procedures and is more common in adults [4]. In our case, the primary etiology was mechanical external pressure during a two-week ICU stay for Guillan-Barré syndrome. Systemic factors common in critical ICU scenarios, such as tissue hypoperfusion, can globally compromise cellular viability [8]. This prolonged ischemic event triggers an inflammatory response affecting hair follicle homeostasis, which ultimately accounts for the development of permanent scarring in the center of the lesion [4]. The duration of pressure is more significant than its intensity, and aggravating factors include the Trendelenburg position, obesity, psychiatric disorders, hypoperfusion, and acidosis [8]. Recently, a case was reported in a child following the use of a gel horseshoe head pad, with the open portion facing towards the top of the head for almost seven hours [9].

In the pediatric population, the clinical profile of PA presents unique challenges. Most documented cases are confined to the neonatal period, where infants in the ICU are at heightened risk due to a disproportionately large head-to-body ratio and an immature, highly permeable scalp barrier [1]. Outside of this neonatal window, PA is exceedingly rare. Clinically, PA presents as a well-defined alopecic area, most commonly in the vertex or occipital region, and convex areas of the scalp are susceptible to ischemia during sustained positioning [8,9]. In older pediatric patients, due to a larger head-to-body ratio, prolonged immobilization can lead the head to increased flexion, which significantly concentrates weight-bearing pressure on the lower occipital area during supine positioning [1]. When coupled with complete presenting flaccid paralysis due to acute peripheral neuropathies, the lack of protective reflexive micromovements shifts the maximum weight-bearing contact point toward the lower occipital scalp. Physiologically, PA shares an identical chronological and ischemic pathway with traditional pressure ulcers; this deep tissue compression against the mattress mimics the development of a stage 1 or 2 pressure injury, where capillary occlusion triggers rapid ischemic cutaneous necrosis of the highly metabolic hair follicles before affecting the deeper dermal layers [1,10].

The clinical timeline of PA is a cornerstone for diagnosis, with onset typically occurring 3-30 days post-event, with spontaneous hair regrowth often observed within 1 to 4 months. The alopecic patch may be preceded by erythema, edema, erosion, or ulceration and can be painful or tender [1,4,9,10]. In our patient, the development of an erythematous and crusty plaque occurred at five weeks post-immobilization, with follow-up at one month post-treatment demonstrating a permanent, irreversible cicatricial center contrasting with peripheral regrowth. A rigorous differential diagnosis is essential in pediatrics to exclude more common entities such as AA, tinea capitis, and trichotillomania. Trichoscopy is considered a critical tool for guiding the diagnosis, where the absence of widespread yellow dots and coiled hairs helps rule out AA and trichotillomania, respectively [11]. Ravaioli et al. observed distinct trichoscopic features based on the disease phase, with the early phase showing dystrophic hairs, broken hairs, black dots, and yellow dots [12]. In the late phase, approximately 1 month after clinical onset, the findings included regrowing hairs of uniform length and multiple circular hairs; only 1 of their 12 patients developed permanent alopecia.

Our patient’s presentation is consistent with the cases reported in the literature. Neema et al. conducted a comparative study of trichoscopic findings between PA and AA, analyzing six PA cases against 24 AA controls [13]. They found that the occipital region was the most common location of PA. Among their PA patients, three had a history of prolonged ICU stay, and three had undergone surgery. A unique feature identified in PA was the presence of comedone-like black dots, which were observed in 66% of PA cases and none of the AA controls, a key marker that was distinctly present in our patient. Other findings that predominated in PA were black dots and scarring areas, whereas AA was characterized by a predominance of yellow dots and exclamation mark hairs. Miniaturized hairs were found in both conditions.

Khokar et al. documented two pediatric cases following surgery: a 7-year-old patient who developed occipital alopecia six days after a two-week hospitalization and a seven-hour urologic surgery, and a 3-year-old patient who developed alopecia two weeks after a nine-hour procedure and a three-day hospital stay [1]. The three cases share a similar latency period and affect the same topographic area, which is subjected to constant pressure without positional changes.

Histopathology is typically not required for the diagnosis of PA. However, in cases of scarring alopecia, histopathological findings may include fibrosis with an absence of hair follicles, or remnants locked in the catagen phase, occasionally accompanied by lymphocytic inflammatory infiltrate, and foreign body granulomatous reaction may also be observed [14].

Table 1 lists a comparison of pediatric cases of PA in the literature.

**Table 1:** Pediatric cases of PA with relevant data points such as age, clinical evolution, trichoscopic findings, and hair regrowth outcome h, hours; d, days; w, weeks; m, months; y, years; ICU, intensive care unit; EEG, electroencephalogram; NR, not reported

Author (year)	Age (y)	Etiology and Duration of Pressure	Trichoscopic Findings	Hair regrowth Outcome
Thiem et al. (2014) [11]	12	NR	NR	Partial (9 y)
Porriño-Bustamante et al. (2021) [15]	10	ICU hospitalization (3 d)	Yellow dots, follicular plugs, vellus hairs	NR
Bhatt et al.(2004) [16]	11	Vitreoretinal surgery (4.5 h)	NR	Total (6 m)
Leonardi et al. (2008) [17]	14	Orthodontic headgear (4 m)	NR	Partial (2 m)
Premkumar et al.(2013) [18]	12	Orthodontic headgear (5 m)	NR	Partial (6 w)
Ozdemir et al. (2014) [19]	5	Liver transplantation (8 h)	Broken hairs, black dots	Total (1 m)
Ramot et al. (2014) [20]	18	ICU hospitalization (1 m)	Broken/yellow/black dots, vellus hairs, trichoptilosis, hook hairs, atrichia	NR
Khokhar et al. (2015) [1]	3, 7	Urological procedures (7.5 and 9.5 h)	NR	NR
Papaiordanou et al. (2016) [8]	14	Hospitalization (11 d)	Broken hairs, black dots	NR
Kunapareddy et al. (2018) [21]	11	EEG electrodes (4 d)	Exclamation hairs	Partial (6 w)
Tortelly et al. (2020) [22]	2	Hypospadias correction surgery (9 h)	Broken hairs, black dots, vellus hairs	Total (6 w)
Ravaioli et al. (2019) [12]	< 18 (n =6)	NR	Dystrophic hairs, black/yellow dots	Total (91.7% of cases at 3 m)
Sánchez-Tamayo et al. (2026; current case)	4	ICU hospitalization (2 w)	Comedone-like black dots, vellus hairs, absent follicular openings, pili torti, whitish scales	Partial (2 m)

The management of PA is primarily conservative, as spontaneous hair regrowth within three to four months is expected in temporary presentations, though topical corticosteroids and minoxidil have been used [10]. Our patient presented signs of cicatricial alopecia earlier than typically observed. While it has been hypothesized that pediatric patients might exhibit a rapid transition toward scarring due to structural scalp vulnerabilities, this finding appears speculative based on a single case. Thus, it must be cautiously interpreted, as clinical outcomes vary widely depending on exact tissue perfusion and the precise duration of pressure. This suggests a more rapid evolution of the condition in pediatric patients. For further recommendations, more studies are necessary to evaluate the prognosis in pediatric patients with PA and adjust the treatment approach if necessary.

Ultimately, this case highlights the necessity of expanding on risk factors and implementing standardized preventive recommendations linked to established clinical practice guidelines for pressure sores [1,9]. To mitigate the risk of PA, protocols should mandate frequent repositioning of the head, ideally every 2 hours, or as frequently as every 15 to 30 minutes, along with the use of specialized, soft support surfaces, such as high-density foam supports, pillows, or gel pads to distribute the head's weight uniformly. Strict postoperative and ICU monitoring, especially after prolonged interventions, remains vital to detect early signs of ischemia [9,22].

## Conclusions

PA should be highly suspected in pediatric hospitalizations involving prolonged immobilization, even outside the neonatal period. As presented in this patient, trichoscopy is a critical non-invasive tool for distinguishing it from other more common causes like AA and trichotillomania, to differentiate PA from other conditions by identifying key markers, such as comedone-like black dots, and ruling out other common causes of pediatric patchy alopecia. Prophylactic measures, specifically related to patient mobilization, are advised in at-risk areas to prevent the occurrence of this condition. Early clinical suspicion and immediate implementation of prophylactic measures, specifically related to patient mobilization, are essential to mitigate ischemic damage and minimize the risk of potential cicatricial alopecia. Further studies focusing on the long-term prognosis of PA in the pediatric population are required to establish a clearer understanding in this age group.
